# Commuting-Adjusted Short-Term Health Impact Assessment of Airborne Fine Particles with Uncertainty Quantification via Monte Carlo Simulation

**DOI:** 10.1289/ehp.1408218

**Published:** 2014-10-17

**Authors:** Michela Baccini, Laura Grisotto, Dolores Catelan, Dario Consonni, Pier Alberto Bertazzi, Annibale Biggeri

**Affiliations:** 1 Department of Statistics, Informatics and Applications “G. Parenti,” University of Florence, Florence, Italy; 2 Biostatistics Unit, Cancer Prevention and Research Institute (ISPO), Florence, Italy; 3 Epidemiology Unit, Department of Preventive Medicine, Fondazione IRCCS (Istituto Di Ricovero e Cura a Carattere Scientifico) Ca` Granda, Ospedale Maggiore Policlinico, Milan, Italy; 4 Department of Clinical Sciences and Community Health, Università degli Studi di Milano, Milan, Italy

## Abstract

Background: Exposure to air pollution is associated with a short-term increase in mortality, and this field has begun to focus on health impact assessment.

Objectives: Our aim was to estimate the impact of PM_10_ on mortality within 2 days from the exposure in the Italian region of Lombardy for the year 2007, at the municipality level, examining exposure entailed by daily intermunicipality commuting and accounting for uncertainty propagation.

Methods: We combined data from different sources to derive probabilistic distributions for all input quantities used to calculate attributable deaths (mortality rates, PM_10_ concentrations, estimated PM_10_ effects, and commuting flows) and applied a Monte Carlo procedure to propagate uncertainty and sample the distribution of attributable deaths for each municipality.

Results: We estimated that annual average PM_10_ concentrations above the World Health Organization-recommended threshold of 20 μg/m^3^ were responsible for 865 short-term deaths (80% credibility interval: 475, 1,401), 26% of which were attributable to PM_10_ above the European Union limit of 40 μg/m^3^. Reducing annual average PM_10_ concentrations > 20 μg/m^3^ by 20% would have reduced the number of attributable deaths by 36%. The largest estimated impacts were along the basin of the Po River and in the largest cities. Commuting contributed to the spatial distribution of the estimated impact.

Conclusions: Our estimates, which incorporated uncertainty quantification, indicate that the short-term impact of PM_10_ on mortality in Lombardy in 2007 was notable, and that reduction in air pollution would have had a substantial beneficial effect on population health. Using commuting data helped to identify critical areas for prioritizing intervention.

Citation: Baccini M, Grisotto L, Catelan D, Consonni D, Bertazzi PA, Biggeri A. 2015. Commuting-adjusted short-term health impact assessment of airborne fine particles with uncertainty quantification via Monte Carlo simulation. Environ Health Perspect 123:27–33; http://dx.doi.org/10.1289/ehp.1408218

## Introduction

The role of air pollution in short-term and long-term disease causation is widely recognized [International Agency for Research on Cancer 2013; World Health Organization (WHO) 2013]; many health impact assessments have been published, and others are ongoing (Baccini et al. 2011; Ballester et al. 2008; Heal et al. 2013; Künzli et al. 2000; Li et al. 2010; Martuzzi et al. 2006; Medina et al. 2004). Estimates of the short-term impact of air pollution on mortality (i.e., the impact within a limited number of days from the exposure, usually less than a week) have advantages over estimates of long-term impact for assessing the effectiveness of emissions reduction policies, because they are less influenced by latency time, cumulative exposure, and demographic dynamics (Flachs et al. 2013). Moreover, because the prevalence of exposure is high, population-level effects on mortality may be large, despite the small relative risks measured by the epidemiological models.

The treatment of uncertainty arising from different sources is a major point of concern when evaluating the short-term impact of air pollution (Knol et al. 2009). If air pollution level and total number of events are known, only sampling variability around the effect estimate has to be considered (Baccini et al. 2011; Orru et al. 2009). However, exposure and mortality levels are often predicted rather than observed—for example, when information for a specific area and/or time period is lacking. In these situations, approaches that deal simultaneously with different sources of uncertainty are needed.

Uncertainty can be accounted for by using Monte Carlo (MC) techniques that generate samples from unknown output probability distributions based on repeated random sampling from independent known input distributions, particularly when a closed form of the output distribution is impossible or difficult to obtain, as in complex nonlinear or multidimensional problems (Kumamoto and Henley 1996). This makes it possible to incorporate in the output the uncertainty affecting the inputs. MC methods for propagating uncertainty have not been widely applied in the context of environmental health impact assessment. Mesa-Frias et al. (2013) conducted a systematic review of methods used to quantify uncertainty in this field: Only 19 studies between 2000 and 2012 addressed uncertainty; of these, only 14 adopted probabilistic approaches.

A second concern is the assumption of a static (noncommuting) population. Few studies have investigated the influence of daily commuting on population exposure to air pollution (e.g., Berrocal et al. 2011; Calder et al. 2008). Recently, the European Topic Centre on Air Pollution and Climate Change Mitigation evaluated the contribution of population commuting on exposure to particulate matter in European urban areas and stated that exposure estimates that include commuting may be larger than those based on a static assumption because people usually move from lower- to higher-polluted areas (Larssen et al. 2012).

Our aim in this paper was to estimate the short-term impact of high concentrations of particles up to 10 μm in diameter (PM_10_) on mortality in the Italian region of Lombardy in 2007 at the municipality level, considering commuting and accounting for uncertainties that arise in the calculation of attributable deaths (AD) via MC simulation. We focused on PM_10_ because the evidence for the causal mechanism between exposure and health damage is more consolidated for this air pollutant than for others (Anderson et al. 2012).

**Figure 1:**
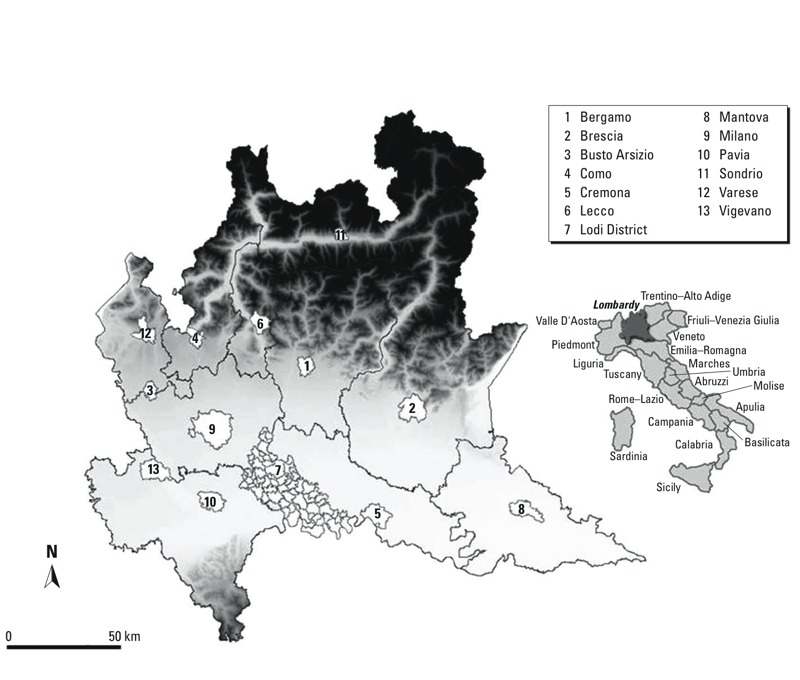
Subdivision of Lombardy by province in 2007. The provincial capitals and the cities of Busto Arsizio and Vigevano are indicated in white. Grayscale expresses altitude, with darker tones indicating mountain areas. Reproduced from Baccini et al. (2011), with permission of Oxford University Press.

*The region of Lombardy*. Lombardy is a 23,865-km^2^ region in northwestern Italy, which can be geographically and economically divided into three zones: the mountain range of the Alps, the sloping foothills, and the highly industrialized and populated basin of the Po River. The latter is characterized by a high level of air pollution due to frequent thermal inversion, with pollution being trapped close to the ground level. In 2007, Lombardy had 9.8 million inhabitants living in 1,546 municipalities located among 11 provinces (Figure 1).

Mobility in Lombardy is substantial, with the highest percentage in Italy (53%) of residents commuting daily to workplaces or schools, half of them out of their residence municipality (Regione Lombardia 2004).

Levels of air pollution vary throughout the region. Between 2003 and 2006 the annual average concentration of PM_10_ in Milan, the capital of the region (1,299,633 inhabitants in 2007), was 52.5 μg/m^3^, with 95% of daily concentrations > 20 μg/m^3^, whereas the average across the other most densely populated areas of Lombardy was 45.4 μg/m^3^ (Baccini et al. 2011).

## Methods

*Data and input distributions*. To derive the probabilistic distributions for input data used in the MC procedure, we used information on total mortality, PM_10_ concentrations, PM_10_ effects, and intermunicipality commuting flows, arising from different sources.

Smoothed crude mortality rates. For each municipality, the average number of deaths during the period 2000–2004 from the Regional Mortality Register and the number of inhabitants at the 2001 Italian population census were available [Italian National Institute of Statistics (Istat) 2014].

Because risk estimates from small areas suffer from substantial variability, we applied a method, widely used in disease mapping, to smooth crude mortality rates at the municipality level (Besag and Kooperberg 1995; Besag et al. 1991). Specifically, we specified a Bayesian model that accounted for structured and unstructured spatial variability, according to the Besag, York, and Mollie’s (BYM) proposal (Besag et al. 1991). Let *deaths_i_* be the total number of deaths during the period 2000–2004 in municipality *i* (*i* = 1, 2,...,1,546). We assumed that *deaths_i_* followed a Poisson distribution with mean given by the product of the death rate *r_i_* and the population size *D_i_*, which we estimated to be five times the population size in 2001. Moreover, we specified a random-effect log-linear model on *r_i_*:

log(*r_i_*) = *u_i_* + *v_i_*, [1]

where *u_i_* and *v_i_* were independent random terms representing the unstructured spatial variability component and the structured spatial variability component, respectively. We assumed that *u_i_* were independent and normally distributed with mean 0 and variance τ*_u_*^2^, and that *v_i_* followed an intrinsic conditional autoregressive (ICAR) model. In other words, for each *S_i_*, the set of the *n_i_* municipalities adjacent to municipality *i*, we assumed the following conditional distribution for *v_i_*:

*v_i_* | *v_j_* ∈ *S_i_* ~ *N*(^^–^^*v_i_,* τ*_v_*^2^/*n_i_*), [2]

where ^^–^^*v_i_* was the mean of *v_i_* for the municipalities belonging to *S_i_*, and τ*_v_*^2^/*n_i_* was their conditional variance (Besag and Kooperberg 1995). Through the random terms *u_i_* and *v_i_*, the BYM model shrinks the crude rate estimates toward both the local and the general mean.

After defining noninformative inverse gamma priors on the variance parameters, we used Monte Carlo Markov Chain (MCMC) methods to obtain a sample from the joint posterior distribution of the smoothed crude rates *r_i_*. The smoothed crude rates reflect spatial variability of mortality, but at the same time are more stable than the observed crude rates, which could be very unstable for the smallest cities. This analysis was performed using WinBUGS 1.4.3 (Lunn et al. 2000).

Predicted annual average concentrations of PM_10._ Data about annual concentrations of PM_10_ were available from two sources: a Eulerian photochemical model developed by the Regional Agency of Environmental Protection (Milan, Italy) that accounted for transport, chemical conversion, and deposition of atmospheric pollutants (Silibello et al. 2008), and the regional monitoring network for air quality control. The Eulerian model provided predictions at the level of 4 × 4 km grid cells that covered the region; predictions were in the form of cell averages and, because of the deterministic nature of the model, did not convey any information about the inherent uncertainty around the prediction process. Data from monitors were sparsely collected from 58 monitors in the region. To obtain an estimate of the uncertainty around the predictions while retaining coverage of the entire region, we used data from both sources to specify a Bayesian geostatistical model, in the form of universal kriging, and used MCMC methods to obtain a sample from the joint posterior predictive distribution of the annual averages at the municipality level.

We assumed that the vector of the log annual average concentrations from the 58 monitors, log(x), was a realization from a multivariate normal distribution with a mean vector μ depending on z*_s_*, the vector of the annual average concentrations predicted by the Eulerian model for the grid cells where the 58 monitors were located, and variance covariance matrix Σ = {Σ*_ij_*} expressing the spatial correlation structure as a function of the distance between pairs of monitors. Specifically, μ = α + βz*_s_*, where α and β were regression coefficients, and Σ*_ij_* = σ^2^*f*(*d_ij_*;κ,φ), where *f*(*d_ij_*;κ,φ) = exp(–φ*d_ij_*κ), σ^2^ was a variance parameter, *d_ij_* was the known distance between monitor *i* and monitor *j*, and φ and κ were positive parameters (κ *≤* 2) that controlled the rate of decline of the correlation by distance and the amount of spatial smoothing, respectively. Priors for φ and κ were chosen to produce zero correlation between any pair of points at the maximum distance (250 km) and one correlation at the minimum distance (3 km); uninformative priors were specified for all other parameters in the model.

We applied MCMC methods to obtain a sample from the joint posterior distribution of the model parameters and, using as covariate values z the predictions from the Eulerian model in each 4 × 4 km cell, we obtained a sample from the joint posterior predictive distribution of the annual average levels of PM_10_ at the cell centroids of the entire grid. Finally, we derived the joint posterior predictive distribution for the annual average concentration of PM_10_ for each municipality by integrating over these predicted cell values.

We confirmed the validity of the geostatistical model through leave-one-out cross-validation. Root mean square error (RMSE) and fractional bias (FB) values, evaluated on the log scale, were small (RMSE = 0.010; FB = 0.035%), indicating good predictive performance of the model. This analysis was performed using WinBUGS 1.4.3 (Lunn et al. 2000).

Estimates of PM_10_ effect. Estimates of PM_10_ effect were derived from the Bayesian random effects meta-analysis in Baccini et al. (2011), which combined estimates of the effect of PM_10_ during the same day and previous day (lag 0–1) on mortality in 13 areas in Lombardy during 2003–2006. The 13 areas were the entire administrative agricultural district of Lodi; the provincial capitals of Bergamo, Brescia, Como, Cremona, Lecco, Mantova, Milano, Pavia, Sondrio, and Varese; and the large municipalities of Busto Arsizio and Vigevano. For the present impact evaluation we used the joint posterior distribution of the city-specific effects for the 13 areas included in the meta-analysis, and the posterior predictive distribution of a generic city-specific effect for all other municipalities (Riley et al. 2011). A sample from these distributions was obtained using MCMC methods.

Probability of commuting. Data on regular commuting flows over the region were derived from the 2001 Italian population census (see Supplemental Material, Figure S1). For each municipality *i*, we knew the absolute number of inhabitants that regularly commuted to school or work in municipality *j* (*c_ij_*, *j* ≠ *i*). Specifying a vague *a priori* beta distribution on the commuting probability from municipality *i* to *j* (*p_ij_*), and a binomial likelihood for *c_ij_* with binomial denominator equal to the population of *i* at the 2001 census (*pop_i_*^2001^), the resulting posterior distribution for *p_ij_* was a beta distribution with parameters 1 + *c_ij_* and 1 + *pop_i_*^2001^ – *c_ij_*. We assumed that commuting probabilities were mutually independent. Because of the small size of the commuting probabilities, no constraint was needed in practice to assure that, once *i* was fixed, the sum of all *p_ij_* (where *j* ≠ *i*) was < 1.

*AD calculation*. Let us assume that the exposure is homogeneous within the municipality; in the absence of intermunicipality commuting, the fraction of deaths among residents of municipality *i* that are attributable to PM_10_ depends only on the exposure–response curve and the concentration of PM_10_ in municipality *i*. Then, if one assumes linearity on a log scale, the deaths attributable to exposures exceeding the threshold *x*_0_ (AD*_i_*) can be calculated for each municipality as

*AD_i_* = *y_i_* – *y_i_*/exp[β*_i_* × (*x_i_* – *x*_0_) × *I*(*x_i_* > *x*_0_)], [3]

where *y_i_* is the total number of deaths in municipality *i*, β*_i_* is the estimated PM_10_ effect in *i*, *x_i_* is the annual average PM_10_ concentration in *i*, and *I*(*x_i_* > *x*_0_) is a indicator function with *I* = 1 for *x_i_* > *x*_0_ and *I* = 0 otherwise. Usually *y_i_* is assumed to follow a Poisson distribution with mean *μ_i_* = *pop_i_* × *r_i_,* where *r_i_* is the crude mortality rate and *pop_i_* represents the person-years at risk in municipality *i*, which roughly correspond to the total number of inhabitants in *i* during the year of interest.

In commuting, individuals can be exposed to different levels of PM_10_ during the day, so that Equation 3 is no longer valid. Therefore, for each municipality *i* we calculated three different quantities: *A_i_*, the number of deaths among residents of *i* attributable to exposure in *i*; *B_i_*, the number of deaths among residents of *i* attributable to exposure in *j* (where *j* ≠ *i*); and *C_i_*, the number of deaths among non-residents of *i* attributable to exposure in *i*.

Assuming that, on average, regular commuters spend one-third of their time in municipality *j* (where they work or study), and two-thirds of their time in municipality *i* (where they live),

*A_i_* = *y_i_^S^* – *y_i_^S^*/exp[β*_i_* × (*x_i_ – x*_0_) × *I*(*x_i_* > *x*_0_)], [4]

where *y_i_^S^* is the total number of deaths among the residents in municipality *i* associated with the person-years at risk in *i* [with the superscript *S* indicating the static (noncommuting) portion of the population]. To account for intrinsic variability related to random variation of deaths count, we assumed that *y_i_^S^* followed a Poisson distribution, with mean *μ_i_^S^* equal to the product between person-years at risk and the crude mortality rate *r_i_*:

*μ_i_^S^* = [*pop_i_* – 1/3 × Σ*_j_*_≠_ *_i_ exit*(*i*,*j*)] × *r_i_*, [5]

where *pop_i_* was the intercensual estimate of the population of *i* in 2007 (Istat 2014), and *exit*(*i*,*j*) was the number of individuals who regularly commuted from *i* to work or school in *j* (where *j* ≠ *i*). In turn, *exit*(*i*,*j*) was assumed to follow a binomial distribution with a probability of success equal to the probability of commuting from *i* to *j*, and the number of trials equal to *pop_i_*. Because the probabilities of commuting were small relative to the probability of not commuting, no constraint was needed to avoid negative values of *μ_i_^S^*.

*B_i_* is the sum over *j*, with *j* ≠ *i,* of deaths among residents of *i* attributable to the exposure in *j*, which were estimated based on the person-years at risk spent in municipality *j* by residents of *i*, the crude mortality rate for municipality *i* (*r_i_*), and the attributable risk associated with exposure in *j*:

*B_i_* = Σ*_j_*_≠_ *_i_* {*y_ij_^C^* – *y_ij_^C^*/exp[β*_j_* × (*x_j_ – x*_0_) × *I*(*x_j_* > *x*_0_)]}, [6]

where *y_ij_^C^* is the total number of deaths among commuters from *i* to *j* (with the superscript *C* denoting the commuting portion of the population), assumed to follow a Poisson distribution with mean *μ_ij_^C^* = 1/3 × *exit*(*i*,*j*) × *r_i_.*

Finally, *C_i_* is the sum over *j*, with *j* ≠ *i,* of deaths among commuters from *j* to *i* attributable to exposure in *i*, which were estimated based on the person-years at risk spent in municipality *i* by residents of *j*, the crude mortality rate for municipality *j* (*r_j_*), and the attributable risk associated with exposure in *i*:

*C_i_ =* Σ*_j_*_≠_ *_i_* {*y_ji_^C^* – *y_ji_^C^*/exp[β*_i_* × (*x_i_*–*x*_0_) × *I*(*x_i_* > *x*_0_)]}, [7]

where *y_ji_^C^,* the total number of deaths among commuters from *j* to *i*, was assumed to follow a Poisson distribution with mean *μ_ji_^C^* = 1/3 × *exit*(*j*,*i*) × *r_j_*. In turn, the number of individuals who regularly commuted from *j* to work or school in *i*, *exit*(*j,i*), was assumed to follow a binomial distribution with the probability of success equal to *p_ji_* and the number of trials equal to *pop_j_.*

As the formulas show, we assumed that the PM_10_ effect depends on the municipality where the individual is exposed, whereas the mortality rate is that of the municipality where the individual resides.

By combining *A_i_*, *B_i_*, and *C_i_*, we estimated two impact measures:

*AD_i_^A^* ^+^ *^B^* = *A_i_* + *B_i_*, the number of deaths among residents of *i* attributable to exposures in the region (i.e., the exposure in *i* or in other municipalities of the region); and
*AD_i_^A^* ^+^ *^C^* = *A_i_* + *C_i_*, the number of deaths among residents of the region (i.e., the residents in *i* or in other municipalities of the region) attributable to exposure in *i*.

It should be noted that Σ*_i_B_i_* = Σ*_i_C_i_*, which is the total estimated number of deaths due to commuting-related exposure in the region, and Σ*_i_AD_i_^A + B^* = Σ*_i_AD_i_^A + C^*.

Air pollutant reduction scenarios. We estimated AD under different reduction scenarios (RS) corresponding to different definitions of the threshold *x*_0_:

RS0: *x*_0_ = 20 μg/m^3^, the WHO Air Quality Guideline threshold for PM_10_ annual average (WHO 2005);
RS1: *x*_0_ = 40 μg/m^3^, the European Union (EU) limit for PM_10_ annual average (EU 2008);
RS2: *x*_0_ equal to a reduction of 20% in the observed annual concentration of PM_10_, provided it is > 20 μg/m^3^;
RS3: *x*_0_ equal to a reduction of 20% in the observed annual concentration of PM_10_, provided it is > 40 μg/m^3^.

The RS2 and RS3 scenarios define intermediate targets coherent with realistic policies of progressive reduction in PM_10_ concentration until the limits of 40 μg/m^3^ or 20 μg/m^3^ are reached (EU 2008).

Under RS0 and RS1, for the 11 provinces and their capitals, we also calculated the attributable community rates (ACRs), which allow impact comparison among different populations (Wacholder 2005). ACR is defined as the difference between overall crude risk in the population and risk in the unexposed individuals; in our context, ACR was estimated as the ratio between AD and population size.

*Monte Carlo method*. After obtaining 1,000 independent realizations from the distribution of each input quantity (“Data and input distributions” section; see also Supplemental Material, Table S1, for a summary description of the input distributions), we combined them accordingly to Equations 4–7 to obtain 1,000 values from the joint posterior distribution of all quantities of interest (*A_i_*, *B*_i_, *C_i_*, *AD_i_^A + B^, AD_i_^A + C^*; *i* = 1,2,…,1,546).

MC simulation was performed using R statistical software (R Core Team; http://www.R-project.org/). The code is available at http://www.biostatistica.net/sites/MC_mobility/prog_data.zip.

Evaluating the role of commuting.For each municipality we calculated the logarithm of the ratio between the posterior median of *C_i_* and the posterior median of *B_i_*, as a measure of the balance between AD “exported” (deaths among individuals residing elsewhere in the region attributable to the air pollution level in the municipality *i*) and AD “imported” (deaths among the residents in the municipality *i*, attributable to their commuting-related exposure elsewhere in the region). Negative values of the ratio indicated that the imported AD exceeded the exported AD. Positive values indicated that the exported AD exceeded the imported AD.

The problem of sampling negative values of air pollutant effect. Impact measures are appropriate when dealing with risk factors that are causally related to the outcome, and under these circumstances, negative estimates are structurally impossible. Therefore, we set AD = 0 whenever a negative value for the PM_10_ effect was sampled during the MC procedure, because we were interested in the AD distribution conditional on rejection of the null hypothesis of no PM_10_ effect in favor of the unilateral alternative hypothesis that exposure increased the risk of death.

To obtain conservative estimates avoiding an overestimation of impact, we summarized AD distributions using the median instead of the mean. We also provided the 80% credibility interval (CrI), defined as the 10th and 90th percentiles of the posterior distribution, and the posterior probability of a positive number of AD.

## Results

**Figure 2:**
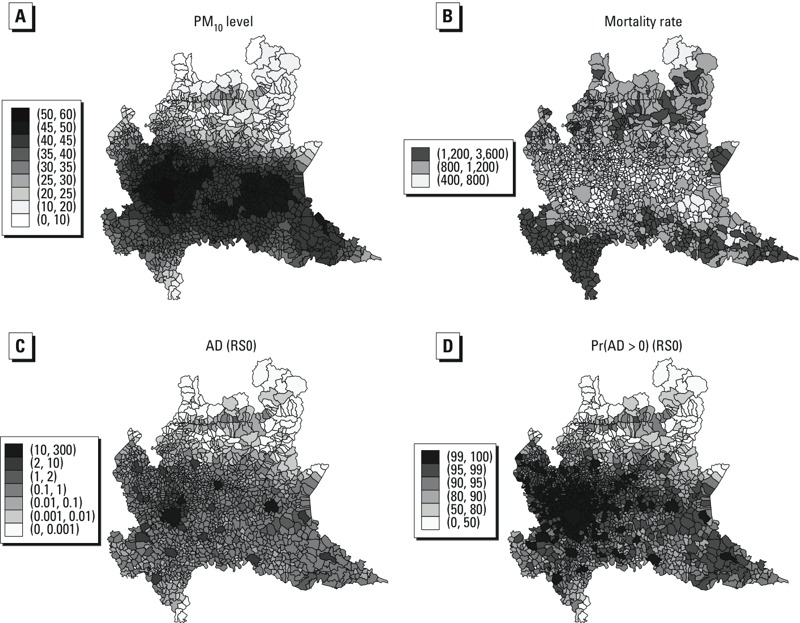
Posterior means of annual average PM_10_ concentrations (μg/m^3^) (*A*) and smoothed crude mortality rates (per 100,000 residents) (*B*); posterior medians of attributable deaths among residents (*C*) and posterior probabilities (%) of a non-null impact (*D*) under the RS0 scenario (for annual average PM_10_ > 20 μg/m^3^), by municipality.

The highest annual average concentrations of PM_10_ were in the central area of the region (Figure 2A). The air pollution level in the municipalities around Milano and Brescia was clearly > 40 μg/m^3^. Milan and the neighboring municipalities were characterized by annual average concentrations > 50 μg/m^3^.

The smoothed crude mortality rates reflected the age distribution of the resident population, with higher estimated rates in municipalities with a larger percentage of elderly people (Figure 2B). Mortality estimates were lower in the central part of the region, except for the three largest cities: Milan, Bergamo, and Brescia.

Estimates of the short-term effect of PM_10_ were similar among cities except for Milan, where the estimated effect was twice the overall meta-analytic estimate (Baccini et al. 2011). The probability of a negative value for the percent variation was close to 0 for Milan, but > 30% for Brescia and Lecco, with the other municipalities in between (see Supplemental Material, Figure S2).

The average percentage of commuters by residence municipality was around 33%, with lower values in the provincial capitals (only 7% in Milan), which, on the contrary, catalyzed entry flows (see Supplemental Material, Figure S1).

Estimated impacts by province of residence are reported in Table 1 as the posterior medians of *AD^A + B^* and 80% CrIs. During 2007, we estimated that under the RS0 scenario there were 865.3 (80% CrI: 475.3, 1400.6) deaths attributable to annual levels of PM_10_ > 20 μg/m^3^ in the region as a whole, and that under the RS1 scenario, approximately 26% of these deaths (AD = 224.9; 80% CrI: 110.6, 367.8) could have been avoided by reducing the annual average of PM_10_ to the EU limit of 40 μg/m^3^. The largest number of AD was estimated for the province of Milan [495.1 for the 20 μg/m^3^ limit (RS0) and 157.6 for the 40 μg/m^3^ limit (RS1)], followed by the provinces of Bergamo and Brescia. The smallest estimated impact was for the province of Sondrio, which is located in the mountain area, with only 3.1 deaths (80% CrI: 0.8, 5.9) under RS0, and a nearly null impact under RS1.

**Table 1:** Attributable deaths (AD) among residents that were attributable to local and regional exposure (*AD^A + B^*) under different scenarios by province: posterior medians and 80% CrIs.

Province	RS0	RS1	RS2	RS3
Bergamo	63.7 (24.4, 116.2)	10.1 (3.5, 19.9)	24.9 (9.8, 45.6)	9.5 (3.2, 18.6)
Brescia	76.2 (27.7, 148.6)	16.8 (5.8, 37.6)	28.0 (10.3, 53.7)	15.0 (5.3, 32.5)
Como	37.4 (14.0, 66.6)	5.7 (2.0, 11.7)	14.7 (5.5, 26.2)	5.3 (1.9, 10.5)
Cremona	31.7 (10.7, 57.7)	5.7 (1.8, 11.7)	11.8 (4.0, 21.7)	5.3 (1.7, 10.8)
Lecco	18.3 (6.6, 32.3)	2.4 (0.8, 4.5)	7.5 (2.6, 13.4)	2.2 (0.8, 4.1)
Lodi	17.9 (6.3, 32.1)	3.0 (1.1, 6.3)	6.7 (2.3, 12.0)	2.8 (1.0, 5.8)
Mantova	31.9 (11.3, 59.3)	4.5 (1.4, 9.2)	12.4 (4.4, 23.0)	4.3 (1.4, 8.5)
Milano	495.1 (286.3, 734.1)	157.6 (71.8, 269.8)	167.0 (96.7, 244.9)	132.7 (65.2, 207.4)
Pavia	43.1 (15.8, 78.6)	4.2 (1.6, 9.7)	18.0 (6.4, 32.2)	4.0 (1.5, 9.3)
Sondrio	3.1 (0.81, 5.9)	< 0.1 (0, 0.1)	1.9 (0.5, 3.6)	< 0.1 (0, 0.1)
Varese	49.4 (18.1, 87.7)	5.7 (2.1, 11.4)	21.3 (7.8, 37.5)	5.3 (1.8, 10.6)
Total	865.3 (475.3, 1400.6)	224.9 (110.6, 367.8)	311.4 (167.3, 506.2)	189.4 (98.1, 304.1)
Abbreviations: CrI, credibility interval; RS0, estimated AD due to annual average PM_10_ > 20 μg/m^3^; RS1, estimated AD due to annual average PM_10_ > 40 μg/m^3^; RS2, estimated AD assuming a 20% reduction in PM_10_ > 20 μg/m^3^; RS3, estimated AD assuming a 20% reduction in PM_10_ > 40 μg/m^3^.				

We estimated that applying a 20% reduction of the annual average PM_10_ concentration in areas where the annual average was > 20 μg/m^3^_,_ or a lower reduction if sufficient to reach the limit of 20 μg/m^3^ (RS2 scenario), would have prevented 311.4 (80% CrI: 167.3, 506.2) deaths in the region (Table 1). Applying a 20% reduction to reach up to 40 μg/m^3^ in areas where the annual average was > 40 μg/m^3^ (scenario RS3) would have prevented 189.4 (80% CrI: 98.1, 304.1) deaths, corresponding to 84% of the total estimated burden of mortality attributable to PM_10_ above the 40 μg/m^3^ EU limit under scenario RS1.

The province with the smallest estimated percentage of municipalities characterized by a non-null impact was the province of Sondrio (59.6% of municipalities with a positive estimated value for *AD_i_^A + B^* under RS0, 6.3% under RS1), whereas the province of Milan had the largest percentage (98.7% and 97.8% under RS0 and RS1, respectively) (see Supplemental Material, Table S2).

In all provinces except Sondrio, we estimated ACRs of > 5 AD per 100,000 inhabitants due to PM_10_ > 20 μg/m^3^ (RS0), with a maximum of 12.7 in the province of Milano (Table 2). The estimated ACR for the entire Lombardy region was 9.1 per 100,000 inhabitants, but in the urban context of the capital cities the ACR reached 15.4/100,000. In fact, with few exceptions, ACRs were higher in provincial capitals than in the provinces as a whole. We estimated that PM_10_ > 40 μg/m^3^ (RS1) resulted in 2.4 deaths per 100,000 inhabitants in the Lombardy region, and 4.4 in the provincial capitals.

**Table 2:** Attributable community rate (ACR) per 100,000 inhabitants under two scenarios by province of residence and in the capital of each province: posterior medians.

Province	Population (*n*)		ACR under RS0		ACR under RS1	
	Province	Capital	Province	Capital	Province	Capital
Bergamo	1,044,820	115,645	6.1	8.9	1.0	0.7
Brescia	1,195,777	190,044	6.4	6.3	1.4	1.3
Como	572,441	83,265	6.5	8.6	1.0	0.3
Cremona	350,368	70,883	9.0	8.7	1.6	0.6
Lecco	327,510	47,006	5.6	4.2	0.7	0.1
Lodi	215,386	42,737	8.3	9.3	1.4	0.9
Mantova	397,533	47,810	8.0	9.8	1.1	1.0
Milano	3,884,481	1,303,437	12.7	20.4	4.1	6.6
Pavia	521,296	70,678	8.3	9.1	0.8	0.5
Sondrio	180,429	21,978	1.7	4.8	< 0.1	< 0.1
Varese	855,400	82,216	5.8	5.9	0.7	0.1
Total	9,545,441	2,075,699	9.1	15.4	2.4	4.4
Abbreviations: RS0, estimated AD due to annual average PM_10_ > 20 μg/m^3^; RS1, estimated AD due to annual average PM_10_ > 40 μg/m^3^.						

The larger cities belonging to the basin of the Po River were characterized by the largest estimated impacts in the region (Figure 2C). The posterior probability of a non-null impact was large in the Milan and Brescia areas and in other urban centers of the Po valley (Figure 2D). The estimated impacts in Milano and Brescia stood out even when the less stringent limit of 40 μg/m^3^ was considered (see Supplemental Material, Figure S3C).

The probability that the number of exported AD (i.e., the deaths among nonresidents of *i* attributable to PM_10_ exposure in *i*; *C_i_*) was greater than the number of imported AD (deaths among residents of *i* attributable to exposure outside of *i*; *B_i_*), was > 60% for all capital cities, except for Lecco and Lodi, where it was around 50% (Table 3). However, the absolute differences between *AD_i_^A + C^* and *AD_i_^A + B^* were always < 1, except for Milan. We estimated 265.6 (80% CrI: 143.8, 414.9) deaths among residents of Milan attributable to PM_10_ exposure anywhere in the region, and 283.8 (80% CrI: 152.1, 443.7) deaths among residents of the region attributable to PM_10_ exposure in Milan, 22.3 (80% CrI: 12.1, 34.7) of which were among individuals residing outside of the capital (*C_i_*).

**Table 3:** Estimated values of *AD^A + B^* and *AD^A + C^* in the provincial capitals under the RS0 scenario: posterior medians, 80% CrIs, and probabilities that the “exported” attributable deaths exceed the “imported” ones.

City	*AD*^*A + B*^ (80% CrI)	*AD*^*A + C*^ (80% CrI)	Pr(C > B)
Bergamo	10.2 (0.5, 23.4)	10.8 (0, 25.3)	0.707
Brescia	12.0 (0.3, 36.5)	12.6 (0, 39.7)	0.608
Como	7.2 (0.3, 15.4)	7.5 (0, 16.5)	0.668
Cremona	6.2 (0.1,14.2)	6.5 (0, 14.9)	0.660
Lecco	2.0 (0.1,5.7)	2.1 (0, 6.2)	0.523
Lodi	4.0 (0.3, 8.5)	4.0 (0, 8.8)	0.494
Mantova	4.7 (0.1, 10.7)	5.1 (0, 12.0)	0.760
Milano	265.6 (143.8, 414.9)	283.8 (152.1, 443.7)	0.996
Pavia	6.4 (0.4, 15.6)	6.9 (0, 17.3)	0.668
Sondrio	1.0 (< 0.1, 2.6)	1.2 (0, 3.0)	0.758
Varese	4.9 (0.3, 12.0)	5.1 (0, 12.9)	0.645
Abbreviations: CrI, credibility interval; RS0, estimated AD due to annual average PM_10_ > 20 μg/m^3^; *AD*^*A + B*^, deaths among city residents attributable to PM_10_ exposure in the city or anywhere in the region; *AD*^*A + C*^, deaths among residents of the entire region attributable to the exposure in the city; Pr(C > B), probability that *C*_*i*_ (“exported” attributable deaths among non-residents due to exposure in city *i*) is larger than *B*_*i*_ (“imported” attributable deaths among residents of city *i* due to exposure outside of city *i*).			

**Figure 3:**
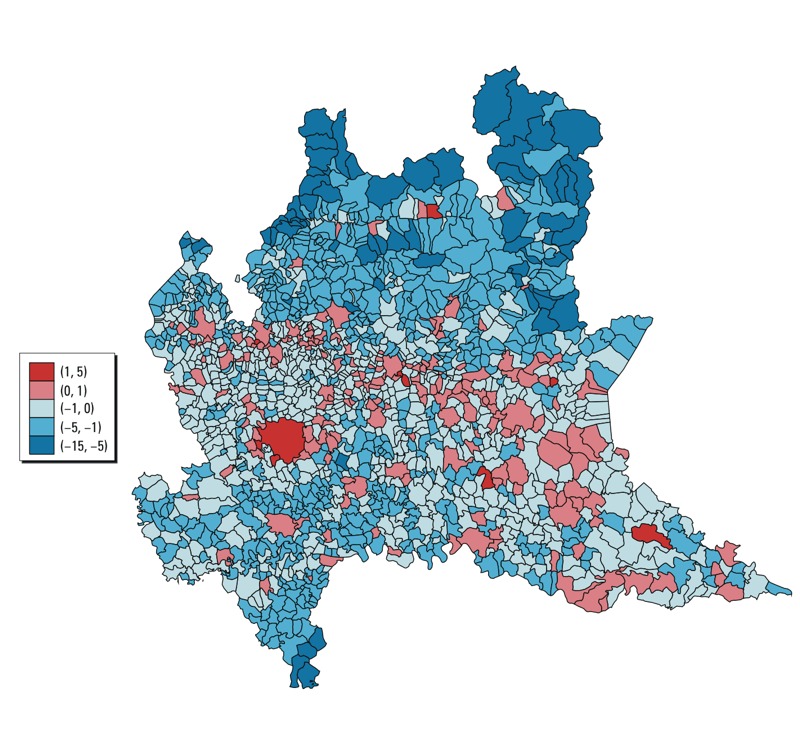
Log ratio between the posterior median of “exported” attributable deaths (*C_i_*, attributable deaths among nonresidents due to exposure in city *i*) and the posterior median of the “imported” attributable deaths (*B_i_*, attributable deaths among residents of city *i* due to exposure outside of city *i*), by municipality.

Figure 3 shows the log ratio between the posterior median of *C_i_* and the posterior median of *B_i_*. The basin of the Po River was characterized by an overall balance between the two estimated flows, except for the cities of Milan, Brescia, Pavia, Cremona, and Mantova, where the ratio was large and positive, indicating that these cities tended to “export” impact elsewhere in the region rather than to “import” it. In contrast, our estimates suggest that the southern part of the Pavia province and the municipalities of the Alpine area, except Sondrio, tended mainly to “import” impact from the rest of the region.

## Discussion

Our analysis indicates that a 20% reduction in annual average PM_10_ concentrations > 40 μg/m^3^ would have substantially reduced the short-term impact of PM_10_ exposures on population mortality in the Lombardy region in 2007. The largest estimated impacts were in the municipalities along the basin of the Po River and in the largest cities, particularly Milan and Brescia. Around 57% of the total estimated number of deaths attributable to annual average PM_10_ concentrations > 20 μg/m^3^ was in the province of Milan. For PM_10_ > 40 μg/m^3^, this percentage rose to 70%. This is not surprising, because the Po basin is one of the less windy areas in Europe, with poor rainfall and frequent thermal inversions that trap pollutants close to the ground. In addition, the areas around Milano and Brescia are the most densely populated and the most industrialized, with tens of thousands of enterprises connected through road transportation.

In estimating the impact of PM_10_ exposures, we relaxed the strong hypothesis of a static population by considering commuting. This enabled us to identify areas where local PM_10_ exposure spread its impact throughout the region (Milan and its hinterland, and other main cities in the basin of the Po River) and municipalities whose residents were primarily impacted by exposure to PM_10_ in the other areas where they worked or attended school (the mountain municipalities). Not considering commuting would have resulted in underestimating the impact of pollution in those areas. Regarding the size of the phenomenon, the estimated number of deaths due to the exposure outside the municipality of residence was sometimes not negligible.

The MC approach allowed us to account for sampling variability resulting from the use of estimated values for PM_10_ effects, PM_10_ concentrations, mortality rates, and commuting probabilities, and for intrinsic variability related to random variation of deaths counts and commuting flows. Moreover, by sampling from the joint posterior distributions of the input quantities, we preserved between-municipality correlations in the output so that reliable CrIs for the total number of AD in the region, and by province, could be derived by summing the estimated impacts over municipalities.

As a consequence of having considered many sources of uncertainty, CrIs were quite large. For example, the total estimated impact for the region ranged from 475.3 to 1400.6 AD with an 80% probability. These large CrIs indicate that one should focus on the whole AD distribution and not only on the point estimate. We reported also the posterior probabilities of AD > 0. The existence of a relevant impact of PM_10_ over the region is strongly supported by the fact that for the most municipalities the probability of a non-null impact was > 90%; restricting the attention to the basin of the Po River, for most municipalities this probability was > 95%.

Individuals who commute from their residence area to work or attend school may be less frail and less susceptible to adverse effects of PM_10_ than the general population; if so, applying mortality rates and effect estimates derived for the general population to commuters could overestimate the commuting-related impact of exposure. We think that, although some degree of overestimation is possible, it may be offset by underestimation of PM_10_ exposures among commuters, who are probably exposed to higher levels of air pollution than noncommuters because they travel along highly polluted “corridors,” and because PM_10_ concentrations during working hours tend to be higher than nighttime concentrations (Larssen et al. 2012). However, it is difficult to quantify these potential biases.

We addressed the issue of intermunicipality commuting, but did not consider intraurban commuting, which would require finer data at the suburban level and imply removing the assumption of homogeneous exposure within municipality. Also, although we did not account for commuting from neighboring regions, we accounted for commuting to them. This could explain unexpected low or high values of the log ratio between *C_i_* and *B_i_* for municipalities situated on the border of the region.

We did not consider “structural uncertainty” related to the model assumptions (Smith 2006). Some of these assumptions are common to analyses of the short-term effect of air pollution (homogeneity of exposure within municipality, log-linear exposure–response relationship), and others are related to AD calculation in the presence of commuting. In particular, we assumed that the mortality rate depends on the municipality where the individual resides, whereas the PM_10_ effect depends on the municipality where the individual is exposed. The first assumption implies that the mortality rate depends on the socioeconomic, demographic, and environmental context where the individual resides. The second assumption implies that the exposure effect depends on local environmental factors that modulate the action of PM_10_ on health (for example, meteorological conditions, PM_10_ composition, or presence of special emission sources). This second assumption does not account for discrepancies among effect estimates that could be related to demographic and socioeconomic factors.

Finally we assumed that commuters spend a third of their time in the municipality where they work or study, supposing an overall balance between time spent traveling and weekend break. Changing this proportion might alter results at the municipality level.

Because our impact assessment refers to 1 year in the past, the epistemic component of the uncertainty related to the incomplete knowledge of future exposures and future sociodemographic conditions did not play a role in this analysis.

## Conclusions

Using data on population daily commuting, we estimated for each municipality the short-term impact of PM_10_ exposure not only on the mortality of residents, but also on the mortality of commuters residing elsewhere in the region, and we identified critical areas where PM_10_ pollution was likely to have the greatest impacts on population health. Our estimates accounted for several sources of uncertainty. Overall, our findings suggest an important impact of PM_10_ exposure on mortality, and also that different scenarios of PM_10_ reduction would have had a substantial beneficial effect, especially considering that we did not consider morbidity attributable to air pollution, which could be considerable. The results of this study can help policy makers prioritize interventions.

## Supplemental Material

(435 KB) PDF
