# Supplementary material for: Commuting-Adjusted Short-Term Health Impact Assessment of Airborne Fine Particles with Uncertainty Quantification via Monte Carlo Simulation
Source: Environ Health Perspect. 2014 Oct 17;123(1):27–33. doi: 10.1289/ehp.1408218 (PMC4286278; doi:10.1289/ehp.1408218)
Supplement: (435 KB) PDF [file ehp.1408218.s001.508.pdf]

*Environmental Health Perspectives* doi:10.1289/ehp.1408218

# Supplemental Material

# Commuting-Adjusted Short-Term Health Impact Assessment of Airborne Fine Particles with Uncertainty Quantification via Monte Carlo Simulation

Michela Baccini, Laura Grisotto, Dolores Catelan, Dario Consonni, Pier Alberto Bertazzi, and Annibale Biggeri

**Table S1.** Input parameters involved in attributable deaths calculation, with a description of the related posterior distributions used in Monte Carlo simulations.

| Input parameters | Input probabilistic distribution |
|---|---|
| $\beta_1, \beta_2, \ldots, \beta_{1546}$ | Joint posterior predictive distribution of the $PM_{10}$ effects by municipality, from Bayesian meta-analysis (Baccini et al. 2011) |
| $r_1, r_2, \ldots, r_{1546}$ | Joint posterior distribution of the smoothed crude mortality rates by municipality, from the BYM model |
| $x_1, x_2, \ldots, x_{1546}$ | Joint posterior predictive distribution of the annual average concentrations of $PM_{10}$ by municipality, from the Bayesian geostatistical model |
| $p_{ij}$ ($i$=1,2,…,1546; $j$=1,2,…,1546; $j \neq i$) | Posterior distribution of the commuting probabilities obtained from independent Beta-Binomial models |

**Table S2.** Estimated percentage[a] of municipalities where there is a positive number of attributable deaths among residents ($AD^{A+B}$), by province and air pollution reduction scenario.

| Province | RS0 | RS1 |
|---|---|---|
| Bergamo | 89.2 | 60.9 |
| Brescia | 83.5 | 65.3 |
| Como | 85.3 | 54.1 |
| Cremona | 91.2 | 77.1 |
| Lecco | 86.8 | 59.9 |
| Lodi | 93.0 | 85.6 |
| Mantova | 93.4 | 73.7 |
| Milano | 98.7 | 97.8 |
| Pavia | 89.1 | 55.5 |
| Sondrio | 59.6 | 6.3 |
| Varese | 92.2 | 49.2 |
| Total | 88.3 | 63.5 |

$AD^{A+B}$: deaths among the residents in the city attributable to the exposure in the city itself and elsewhere in the region.

RS0: Estimated AD due to annual average $PM_{10}$ > 20 µg/m$^3$. RS1: Estimated AD due to annual average $PM_{10}$ > 40 µg/m$^3$.

[a]Mean percentage arising from Monte Carlo simulations.

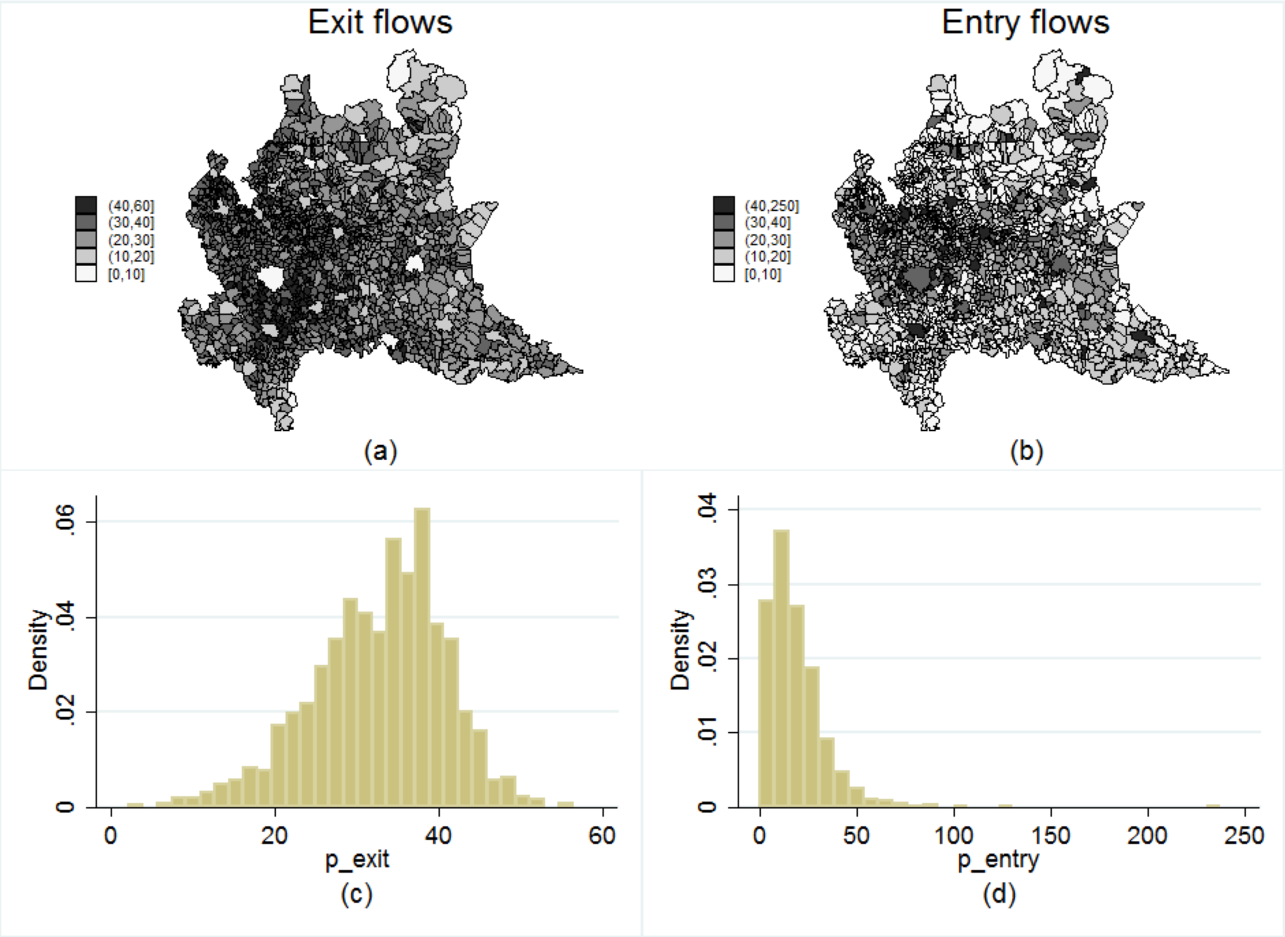


**Figure S1.** Regional map (a) and histogram (c) of the percentage of commuters by municipality of residence (*p_exit*); regional map (b) and histogram (d) of the percentage of commuters by municipality of destination, in respect to the population size of the municipality of destination itself (*p_entry*). Data from the 2001 Italian population census.

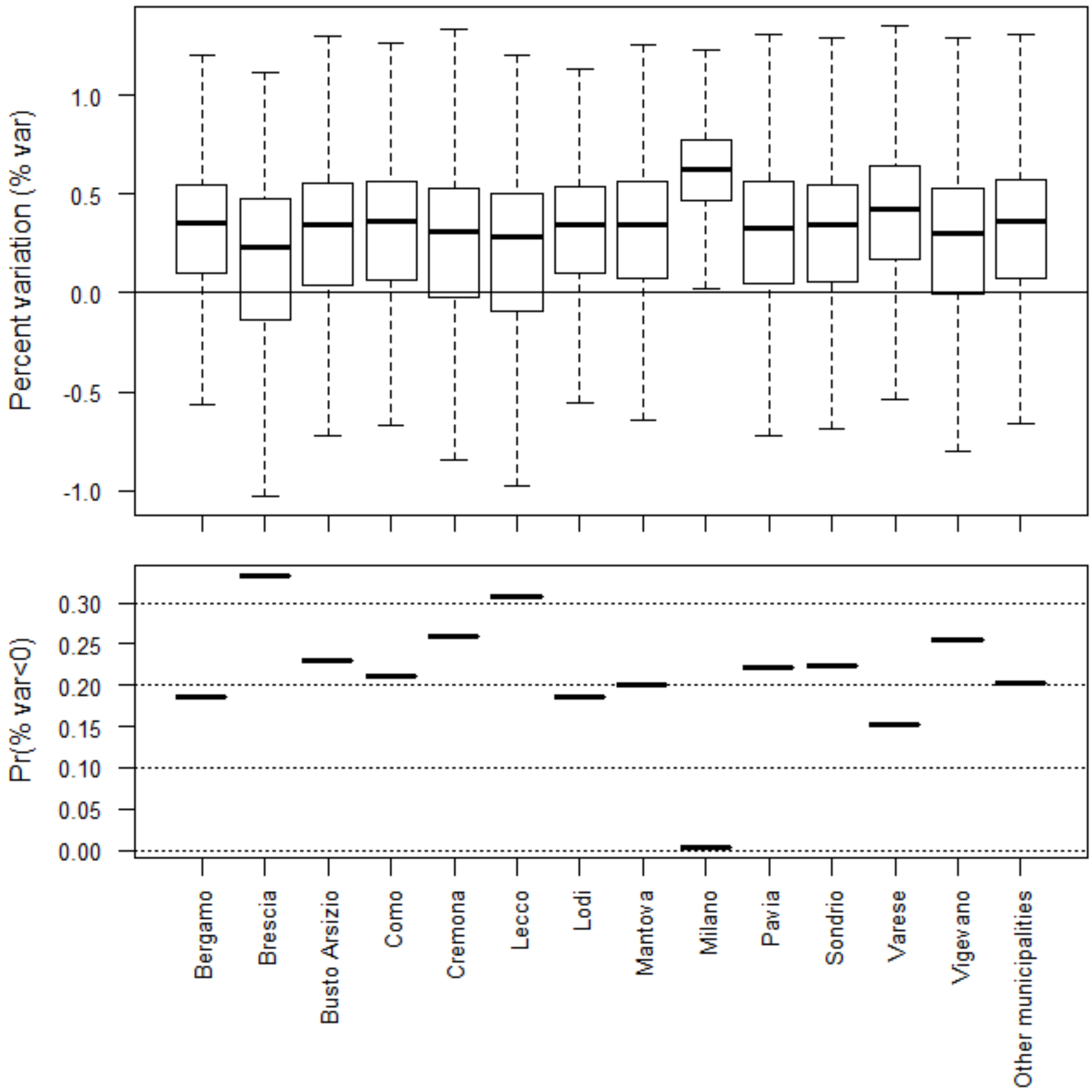


**Figure S2.** City-specific posterior distributions and posterior predictive distribution of the percent variation in total mortality associated to a 10 µg/m$^3$ increase of the average $PM_{10}$ concentration in the current day and in the previous day (upper panel), and posterior probabilities of a negative percent variation (lower panel) (results from Baccini et al. 2011). The predictive posterior distribution expresses the uncertainty around the $PM_{10}$ effect in a city randomly chosen among the 13 included in the meta-analysis. The posterior predictive distribution was obtained by the sum of the overall meta-analytic effect and a random noise from a Normal distribution with variance $\tau^2$, where $\tau^2$ was sampled in turn from the posterior distribution of the heterogeneity among areas. Boxes in the upper panel extend from the 25th to the 75th percentile, horizontal bars represent the median, whiskers extend 1.5 times the length of the interquartile range (IQR) above and below the 75th and 25th percentiles, respectively.

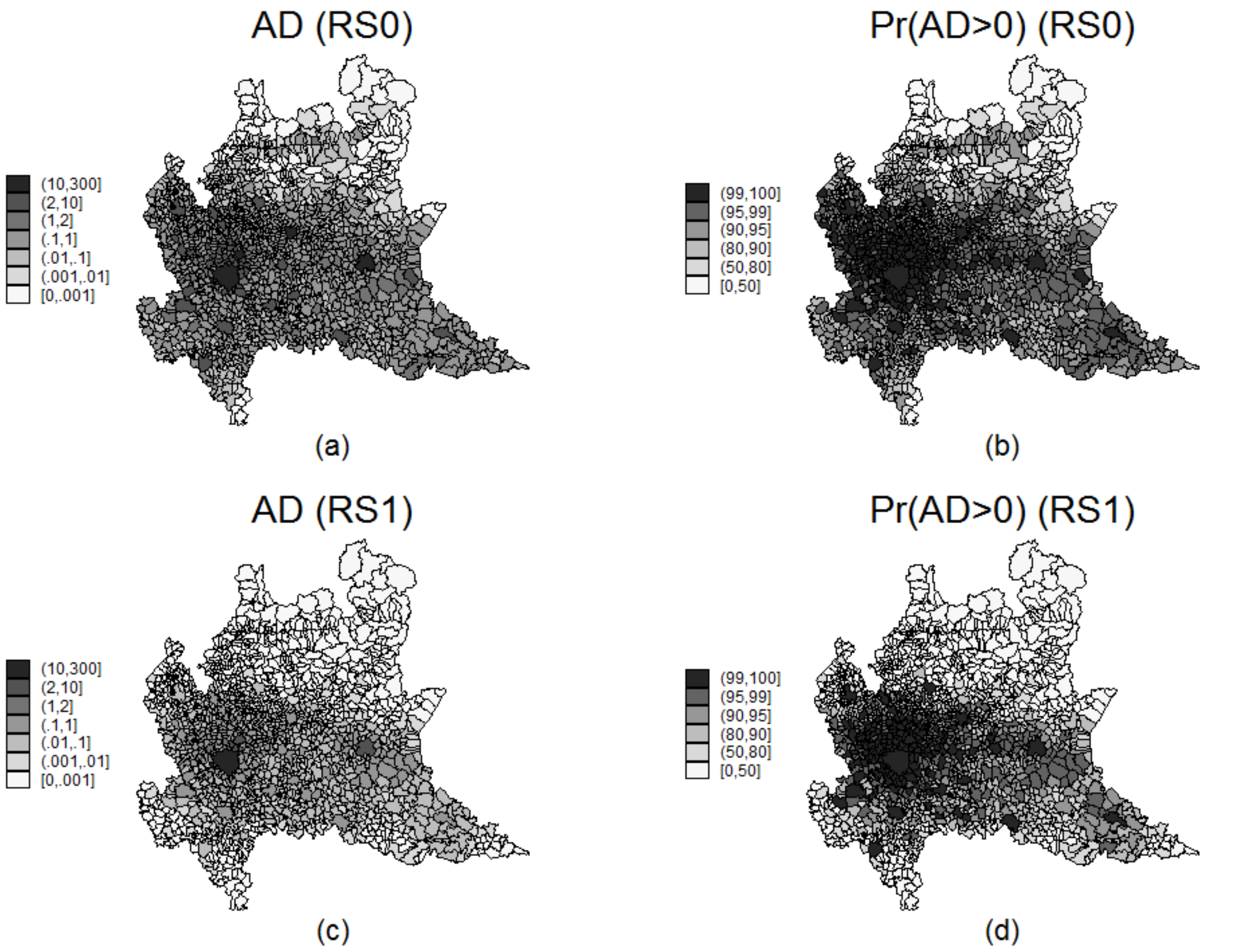


**Figure S3.** Posterior medians of attributable deaths (AD) among residents (a) (c) and posterior probabilities (%) of a non-null impact (b) (d), under the RS0 scenario (for annual average $PM_{10}$ > 20 μg/m$^3$) and under the RS1 scenario (for annual average $PM_{10}$ >40 μg/m$^3$), by municipality.

## Reference

Baccini M, Biggeri A, Grillo P, Consonni D, Bertazzi PA. 2011. Health Impact Assessment of Fine Particle Pollution at the Regional Level. Am J Epidemiol 174:1396-405.
